# Design of wireless battery management system monitoring and automated alarm system based on improved long short-term memory neural network

**DOI:** 10.7717/peerj-cs.1345

**Published:** 2023-05-25

**Authors:** Qingyu Zhang

**Affiliations:** Henan Industry and Trade Vocational College, Henan, China

**Keywords:** Wireless BMS, Socket communication, LSTM neural network, BN algorithm, DAE algorithm, Automated Alarm System

## Abstract

The battery management system (BMS) can intelligently manage and maintain each battery unit while monitoring its status, thereby preventing any possible overcharge or over-discharge of the battery. In BMS research, battery state parameter collection and analysis are essential. However, traditional data collection methods require personnel to be present at the scene, leading to offline data acquisition. Therefore, this study aimed to develop a wireless BMS monitoring and alarm system based on socket connection that would enable researchers to observe the operating parameters and problem details of the battery pack from a distance. A device like this effectively raises the battery’s level of cognitive control. In the study, the researchers first designed the overall scheme of the BMS remote monitoring system, followed by building a wireless BMS monitoring and alarm system. Performance evaluations of the system were then conducted to confirm its effectiveness. A Long Short-Term Memory (LSTM) network enhanced by the Batch Normalization (BN) technique was applied to the time series data of battery parameters to solve the large accuracy inaccuracy in battery state of charge estimate. Furthermore, the Denoise Auto Encoder (DAE) algorithm was utilized to denoise the data and reduce the model’s parameter dependence. The accuracy and robustness of the estimation are improved, and the model error is gradually stabilized within 5%.

## Introduction

With the wide application of new energy electric vehicles, batteries’ capacity, safety, health status and endurance have increasingly become the focus of attention (Zhang et al., 2020; Hua & Dong, 2022). The battery management system (BMS) is a system that monitors and controls the battery, feeds back the collected battery information to users in real-time, and adjusts parameters according to the collected data to give full play to the battery performance (Gabbar, Othman & Abdussami, 2021). A crucial part of large-scale energy storage systems, the battery management system provides data gathering, data transmission, battery condition estimation, equalization, and abnormal warning operations (Ali et al., 2019). It effectively reduces the effects of overcharging, over-discharging, and temperature changes on the performance and life of energy storage batteries, enhance the safety and dependability of the battery pack and lower the cost of energy storage systems’ maintenance. To effectively guarantee safe and stable operation, the BMS monitoring system monitors the battery voltage, current, State of Charge (SOC), battery charging and discharging level, temperature, and other crucial factors in real time (Strominger, 2014).

Considering the system structure design of the battery, AeroVironment Company of the United States developed the Smart Guard system and proposed a highly integrated charging and discharging BMS (MacDougall et al., 2000). Using distributed battery cells allows some battery cells to release energy when the vehicle is running. In contrast, other cells collect power for charging through photovoltaic technology or other methods (Ferguson, Baglino & Kalayjian, 2014). The BATTMAN system designed by Hauck in Germany can be compatible with various battery packs. The management of different battery packs is realized through the combination of software and hardware, dramatically improving BMS’s universality (Wey & Jui, 2013). The BMS produced by Preh has an independent protection chip, which can ensure the safety of the battery pack even if the battery system operates under the limit state  (Garcia et al., 2009).

In all functions of the battery management system, SOC online estimation is at the core. Accurate online SOC estimation can provide a valuable reference for battery pack energy balance and charging and discharging strategies, ensuring the system’s stable operation and prolonging the system’s service life (Zhang et al., 2018). Currently, the research on online estimation methods of battery pack SOC is the current research hotspot, which roughly includes the open circuit voltage (OCV) method, ampere-hour integration method, filtering algorithm, learning algorithm and some hybrid algorithms (Zhou et al., 2021). In the actual use of the battery management system, the central control unit is responsible for SOC online estimation. Therefore, the algorithm complexity and algorithm estimation accuracy should be considered comprehensively when selecting the SOC estimation algorithm to improve the online estimation performance of battery SOC (Hu, Youn & Chung, 2012; Prabhu, Maheswari & Vigneshwaran, 2022).

The above research on the critical technologies of BMS has extensively promoted the development of BMS. However, most traditional monitoring systems are wired, and the wiring is complex. When managing multiple batteries, personnel must debug and set them on-site, making inspection, maintenance and updating quite inconvenient. Once staff leaves the equipment, they cannot monitor and meet the timeliness and mobility requirements of the monitoring system. With the rise of the Internet of Things, cloud platforms, artificial intelligence and other technologies provide ideas for BMS innovation. The Internet of Things technology is used to complete the collection and upload of battery pack-related data, effectively realizing the intelligent collaboration of batteries (Liu et al., 2019). The cloud platform uses virtualization capabilities to transform the data center into a cloud computing architecture. By building a cloud BMS and relying on the cloud platform’s robust storage and computing capabilities, it can achieve relevant functions that cannot be efficiently realized by the terminal and complete wireless monitoring of battery status (Bizeray et al., 2015). Therefore, with the development of artificial intelligence technology, the method of deep learning can be used to estimate the accuracy of battery state of charge, thus breaking through the key technology in BMS design and significantly improving the calculation accuracy and system efficiency.

This article proposes a wireless BMS monitoring and alarm system based on Socket communication and artificial intelligence technology. The main contributions of this article are as follows:

This article designed the overall scheme of the BMS remote monitoring system and built a wireless BMS monitoring and alarm system.

This article proposed an LSTM network improved by the BN algorithm to the time series data of battery parameters to address the problem of significant accuracy error of battery state of charge estimation.

The DAE algorithm is used to denoise and reduce the dependence of the model on parameters.

## Related Works

Currently, the research in BMS mainly focuses on battery state estimation, battery equalization, battery thermal management and other technologies. Among them, the research on battery state primarily focuses on the estimation of battery state of charge (SOC), the prediction of battery state of health (SOH) and the prediction of remaining useful life (RUL).

The online estimation of the battery state of charge is at the core of all the battery management system functions. Shrivastava et al. (2019) believes that accurate online SOC estimation can provide a practical reference for battery pack energy balance and charging and discharging strategies, ensure the system’s stable operation, and prolong the system’s service life. Currently, the research on online estimation methods of battery pack SOC is the current research hotspot, including the open circuit voltage (OCV) method, ampere-hour integration method, filtering algorithm, learning algorithm and some hybrid algorithms (Meng et al., 2017). Among them, the traditional open circuit voltage method is a battery pack SOC estimation method based on offline testing, which requires a small amount of calculation. Still, the battery pack needs a long time to rest fully, so it is difficult to be used for online SOC estimation of the battery pack (Xing et al., 2014). The ampere-hour integration method obtains the charge or discharge quantity of the battery by integrating the current with time. Still, the accuracy of SOC estimation is easily affected by measurement error and noise and will cause a sizeable cumulative mistake. The filtering algorithm can effectively overcome noise interference, measurement error and initial error in battery pack SOC estimation but cannot guarantee numerical stability (Zheng et al., 2018). The learning algorithm requires a large amount of historical data to train the parameters of the SOC estimation model.

As for the estimation of battery health status, there are many definitions of battery pack health status Li, Wang & Gong (2016) conducted experiments on the attenuation rate of power batteries at different ambient temperatures, studied the battery capacity attenuation model with temperature as the accelerating stress, and studied the relationship between the battery capacity retention rate and the working time and ambient temperature. Chen, Fu & Mi (2012) studied lithium-ion battery cells’ SOH evaluation and life prediction methods and proposed a space application example of lithium-ion battery SOH prediction. Lin, Liang & Chen (2012) first introduced the SOH with degraded capacity, established the mapping relationship between AC resistance and ability using support vector regression, proposed to estimate the SOH of two types of HIs, and performed prediction fusion with capacity and internal resistance.

However, the stand-alone battery management implemented only at the device terminals can no longer meet the growing demand for BMS. The BMS implemented by combining technologies such as the Internet of Things and cloud platforms has gradually received extensive attention at home and abroad (Chen et al., 2019). Li et al. (2020) developed a cloud-connected BMS for the car battery pack. The results show that cloud-connected BMS has high accuracy in simulating dynamic battery behavior. Li, Yuan & Wang (2020) proposed a large-scale, cloud-based status monitoring platform for lithium-ion BMS. This research uses Socket communication technology and LSTM neural network to develop a wireless BMS monitoring and alarm system. The platform uses IoT devices and cloud components to realize IoT components, including data collection and wireless communication in the battery module. The results show that more accurate battery status can be obtained by fully using the cloud platform’s storage and computing resources. Nandakumar, Ponnusamy & Mishra (2023) proposed a cloud BMS to improve battery systems’ computing power and data storage capacity through cloud computing. Discusses the application of an equivalent circuit model in the digital twin system of the battery system, which improves the computing power, data storage capacity and reliability of BMS. Baumann, Rohr & Lienkamp (2018) proposed a recursive generation countermeasure network to generate real energy consumption data by learning real energy consumption data, which improved training stability and the quality of generated data. The experiment shows that the data generated by recursive GAN can be used to train the energy prediction model.

Various parameters of the battery are monitored. LSTM neural network is used to estimate the accuracy of the battery charge state so that researchers can remotely view the operating parameters and fault information of the battery pack, and support the subsequent analysis of parameter data, to improve the intellectual control level of the battery effectively.

## Estimation algorithm of battery SOC based on battery operating state

Currently, most algorithms for estimating battery SOC have problems such as a small amount of data and inability to represent all situations, poor model learning ability and estimation accuracy, high parameter dependence and poor robustness. This research is based on many actual battery operation state data, and after data transmission through the Internet of Things technology, the data are labeled and standardized. At the same time, we designed the SOC estimation model of the energy storage battery based on the LSTM depth learning algorithm. We introduced the BN algorithm to optimize and improve the model and used the DAE algorithm to denoise and adapt the model, thus reducing the dependence of the model on parameters and improving the accuracy and robustness of the estimation.

**Figure 1:**
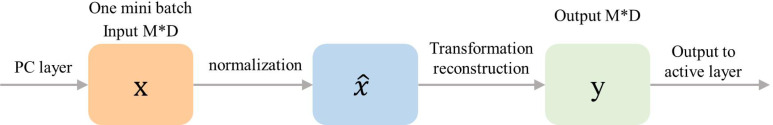
Schematic diagram of the overall structure of the LSTM-DAE model optimized by the DAE algorithm.

DAE adjusts the network structure parameter values of the intermediate nodes of the encoder by performing a back-propagation algorithm on the error term, reduces the reconstruction error through continuous iteration, and finally obtains the result after feature extraction and denoising. Optimized reconstructed input samples to further improve the robustness and adaptability of the SOC prediction model. Based on the LSTM model optimized by the BN algorithm, the DAE algorithm is combined for optimization, and the overall structure of the final LSTM-DAE model is shown in Fig. 1.

Based on the LSTM-BN algorithm, this research uses the DAE algorithm to optimize and improve its network structure and obtain a better LSTM-DAE model. The BN algorithm is used to pull the input value distribution back to the standard global distribution to make the gradient larger and avoid the problem of disappearing gradients. The classification model designs a two-layer network stack LSTM structure whose batch_size is set as 128.

### Network model construction based on LSTM

Neither the traditional Kalman filter for SOC estimation nor the support vector machine regression method considers that SOC, as a battery state, cannot change and often depends on the previous state of charge. As a variant of the recurrent neural network, the LSTM model can effectively deal with nonlinear time series problems and have long-term memory ability. LSTM network has a forgetting gate, an input gate and an output gate, which can input valid or invalid information to its kernel state unit through the threshold.

**Figure 2:**
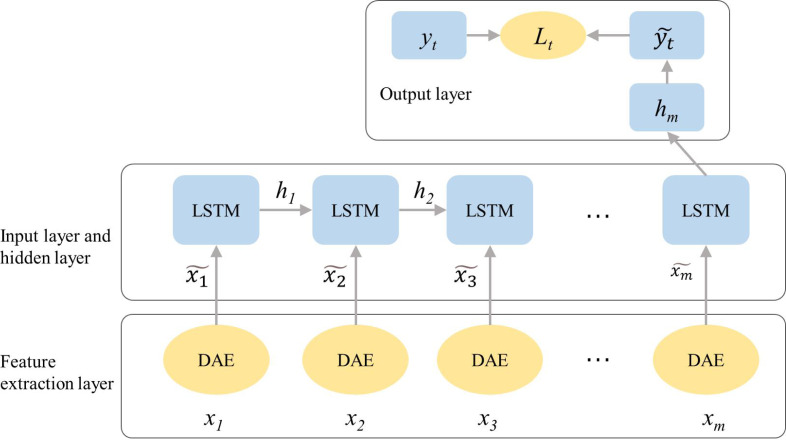
Network structure of SOC prediction model of energy storage battery based on LSTM.

First, by converting the voltage, temperature, current and estimated SOC characteristics of the single battery in the previous state into time series characteristic data, a SOC prediction network based on the short-term memory network LSTM is shown in Fig. 2 is constructed and realized.

According to the operating state of the energy storage battery, such as voltage, current, temperature, etc., collected by BMS in real-time and superimposed with the time characteristics of a moment, an input data format conforming to the timing characteristics is constructed. The size of the matrix is 1*7. After the input data is input into the LSTM network, it first passes through a layer of the LSTM network, and the activation function is RELU. After the dropout layer, it is learned by a layer of LSTM, Then connect the 1*50 dimension fully connected neural network, and through the learning and dimension reduction of the following layers, finally obtain the 1-dimension output value, that is, the predictive value of the energy storage battery SOC.

### Optimization and improvement based on batch normalization algorithm

Although the method of random gradient descent has simplified the training depth network, it still has some drawbacks, such as more severe dependence on the initial set learning rate, parameter initialization, weight attenuation coefficient, dropout ratio and other parameters, so this topic uses BN (batch normalization) algorithm to optimize it.

**Figure 3:**
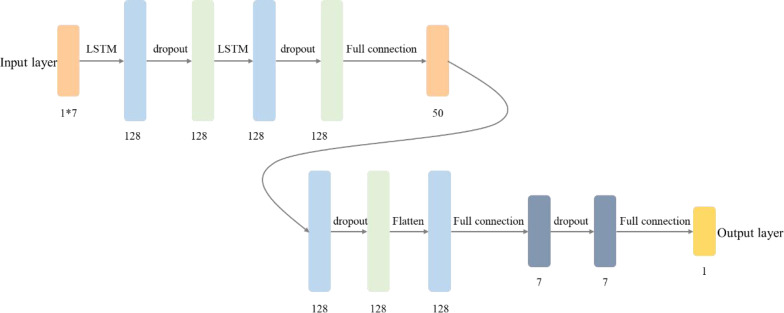
BN algorithm processing flow.

As shown in Fig. 3, the BN algorithm first normalizes the dimensions output after each full connection layer.

(1) \begin{eqnarray*}{\hat {x}}^{ \left( k \right) }= \frac{{x}^{k}-E \left[ {x}^{k} \right] }{\sqrt{var[{x}^{k}]}} (1)\end{eqnarray*}

After the above normalization, to prevent the distribution shift of features, it is necessary to transform and reconstruct the normalized data to restore the original features.

(2) \begin{eqnarray*}{y}^{k}={\gamma }^{k}{\hat {x}}^{k}+{\beta }^{k}(2)\end{eqnarray*}

When ${\gamma }^{\mathrm{k}}=\sqrt{\mathrm{var}[{\mathrm{x}}^{\mathrm{k}}]},{\beta }^{\mathrm{k}}=\mathrm{E} \left[ {\mathrm{x}}^{\mathrm{k}} \right] $, the transformed and reconstructed features are consistent with the original features, by learning two parameters, the network realizes batch normalization, solves the problems of gradient disappearance and gradient explosion, and increases the robustness and error performance of the model.

### Model optimization of network structure combined with Denoising AutoEncoder

To enhance the model’s robustness and stability under complex working conditions, this study proposes to use the Denoising AutoEncoder (DAE) to extract features and denoise the input data. This algorithm is based on the input data to denoise and extract features. The extracted features do not discard some features but fuse and represent them as high-level abstract low-dimensional features that reflect the nature of the data and have more robustness and generalization capabilities.

DAE adds a certain amount of noise to the input data and then realizes the noise removal function through the degradation process in the learning process. The DAE obtains pure noise-free samples for input data with particular noise after encoding, decoding and other operations.

Assume that the input sample data is x ∈ R^n×l^, and DAE randomly sets it to 0 according to a certain proportion, v, to obtain the degenerated input eigenvector $\overline{\mathrm{x}}$. Code them based on degenerate input samples based on linear mapping and the nonlinear activation function to complete the coding phase.

(3) \begin{eqnarray*}\mathrm{h}=\mathrm{g} \left( \mathrm{W}\overline{\mathrm{x}}+{\mathrm{b}}_{1} \right) \end{eqnarray*}

Next, the decoder decodes the encoded feature data to obtain the optimal abstract representation of the input data and complete the decoding phase.

(4) \begin{eqnarray*}\mathrm{z}=\mathrm{g} \left( {\mathrm{W}}^{\mathrm{T}}\mathrm{h}+{\mathrm{b}}_{2} \right) \end{eqnarray*}

In the above formula, z is the reconstruction value of the input data; b_2_ is the node offset term of the decoding layer; g(⋅) is the activation function set by the node.

X and Z are the input training data and the output reconstruction data, respectively. Then the reconstruction error function between the reconstructed data samples and the original input data is:

(5) \begin{eqnarray*}\mathrm{L} \left( \mathrm{X},\mathrm{Z} \right) = \frac{1}{2} \sum _{\mathrm{i}=1}^{\mathrm{m}}{ \left\| {\mathrm{z}}_{\mathrm{ i}}-{\mathrm{x}}_{\mathrm{i}} \right\| }_{2}^{2} \end{eqnarray*}

## Design of Wireless BMS Monitoring and Alarm System

Presently, the informatization degree of BMS monitoring and alarm system in energy management and storage is relatively low. There are high coupling degrees of informatization systems, common system flexibility and scalability, unclear information display, and lack of information on operation data. Related to analysis and utilization, it is impossible to accurately predict the SOC in real time. This research builds a wireless BMS monitoring and alarm system based on Socket communication. This effectively improves the system’s highly flexible and controllable performance and enhances the system’s real-time maintainability and intelligent prediction and early warning protection based on visualization.

The BMS monitoring and alarm system designed in this study adopts the design idea of separating the front and back ends. It is divided into a business, algorithm, communication, and data layer. It has the characteristics of low coupling, high cohesion and high scalability. The corresponding library table is designed, the data storage format is standardized, and the data is intelligently analyzed and applied. Finally, it becomes possible to detect and upload the status of energy storage systems, estimate energy storage battery SOC intelligent algorithms, and visualize energy storage battery operation. Performance and functionality tests were carried out.

### Implementation of the business layer

The business layer of the BMS monitoring and alarm system is mainly responsible for realizing the interaction and request distribution among the system modules, distributing the algorithm business to the algorithm layer, and realizing the corresponding functions by interacting with the data layer modules. This study uses the Spring MVC framework to learn low-coupling system development. Spring Boot is used for automatic configuration, which assists in simplifying the steps of project construction, and uses the Tomcat server as a lightweight application server.

The business layer of this system uses the Spring MVC framework to realize the construction of the business model. It implements a request-driven web framework through the MVC design pattern implemented by Java.

### Implementation of algorithm layer

The algorithm layer preprocesses the request message to extract valuable data features. This system uses Tornado lightweight framework to complete. This technology’s asynchronous, non-blocking IO processing method can use the HTTP protocol to pass parameters to the server, including query strings and data sent in the request body (form data, JSON, XML, etc.), URL regular expression interception, etc. The parameters are preprocessed and transmitted to TensorFlow Serving. The TensorFlow Serving system is well suited to serving multiple models that can dynamically change based on real-world data. It presents a solution to apply the model to actual production. After receiving the parameter data preprocessed by Tornado, this study needs to train and deploy the deep learning algorithm implemented in Chapter 3 to estimate the SOC of the energy storage battery.

The TensorFlow Serving model is mainly used for deploying deep learning algorithm modules, a service system with flexibility, high performance, and use in production environments. New algorithms can be easily deployed through this service system, and multi-model services and distributed APIs can be provided simultaneously. Using the continuous integration framework based on TensorFlow Serving in the algorithm layer is mainly divided into three steps: (1) Model training: including data collection and cleaning, model training, evaluation and optimization; (2) Model online: import the model trained in the previous step into TensorFlow Serving and go online; (3) Service usage: The client communicates with the TensorFlow Serving side through gRPC and RESTful API and obtains the service.

For TensorFlow serving multi-model deployment, you only need to modify the model configuration file to serve the new model. Config, add model_version_policy, place the model file of the new model in the corresponding path, and then start the corresponding service.

### Implementation of the communication layer

**Figure 4:**
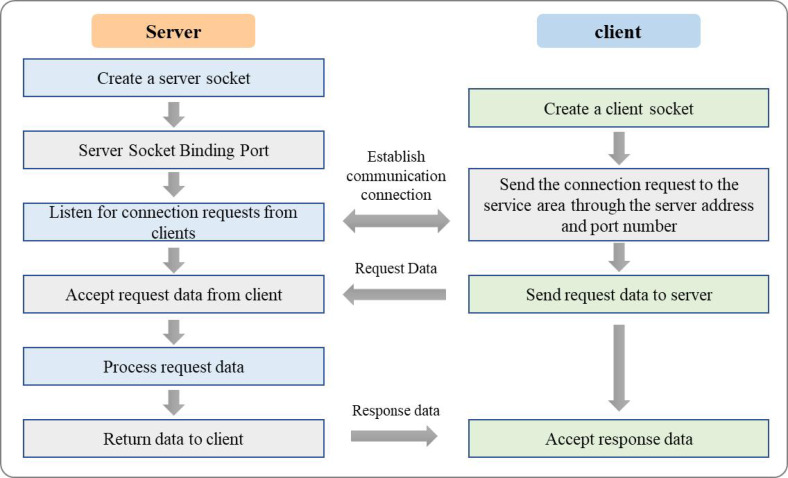
Socket communication process.

Usually, Socket communication consists of two parts: the server side and the client side. The server and client sides can send and receive data from each other by establishing a network connection. The specific process is shown in Fig. 4.

 The Server Socket class is the primary mechanism for implementing server-side TCP/IP communication programs. When a Server Socket object is created, the program provides services on the specified port on the server side and starts listening for possible service requests from clients. When a client requests a connection and is accepted, the server program will create a Socket object to connect with the remote client to implement read-and-write communication operations. The accept() of this class is used to wait and accept a connection from the client. When accept() is called, the server process or thread will be blocked until it listens to a client process that makes a service request and succeeds with its connection. Then accept() will return a newly created server-side Socket object.

### Implementation of the data layer

The data layer is mainly responsible for storing the real-time operation status parameters of the BMS monitoring and alarm system, alarm warning information, user logs and other file data. This research uses the MySQL database management system to save the state parameters of different single cells of the battery pack in separate tables, which increases the operating speed and flexibility of the system. At the same time, the database is mapped and configured using the MyBatis framework under the Spring framework.

**Figure 5:**
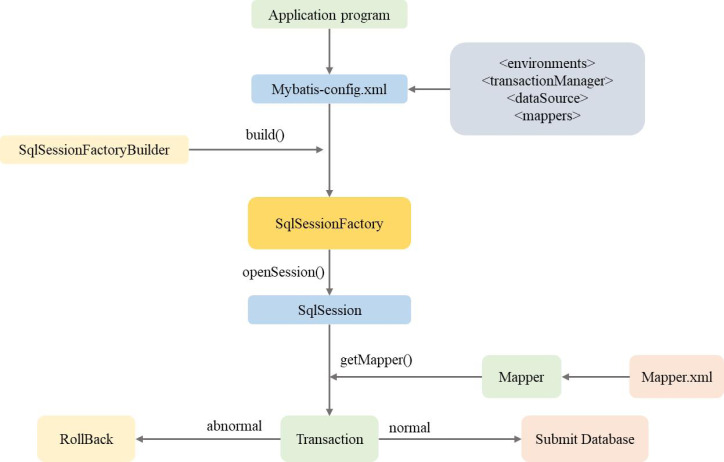
Data layer MyBatis workflow.

This research uses the MyBatis framework to automatically generate the SQL statement that meets the needs by providing the MyBatis mapping method. It can input parameters into the Prepared Statement to achieve automatic input mapping. The query result set can also be mapped to Java objects to meet the output requirements map. Figure 5 displays the workflow. The SqlSession Factory Builder is used by the data layer’s MyBatis application to create the SqlSession Factory from the basis-config.xml configuration file. Then, create a SqlSession instance using a SqlSession Factory instance that was previously constructed. The final step involves making a Mapper object with the SqlSession instance established in the previous step and using the SQL database operation statement that the object has been assigned to complete database operations, such as transaction commit operations or CRUD row operations. Finally, close the SqlSession instance after the procedure is complete.

## Result Analysis and Discussion

This section analyzes the experimental results of the SOC estimation algorithm and the wireless BMS monitoring alarm system.

### Performance analysis of battery SOC estimation algorithm

This study first completed the training based on the extended short-term memory network on the preprocessed data set and obtained the training model LSTM-origin. Afterward, based on batch normalization, the learning and convergence capabilities of the deep learning network were improved and optimized. The model LSTM-BN was obtained by retraining. Finally, the DAE compression autoencoder is used to optimize the generalization ability and robustness of the model, and the model LSTM-DAE is obtained by retraining. Models after training using the same training set are tested for performance metrics on the same tests.

**Figure 6:**
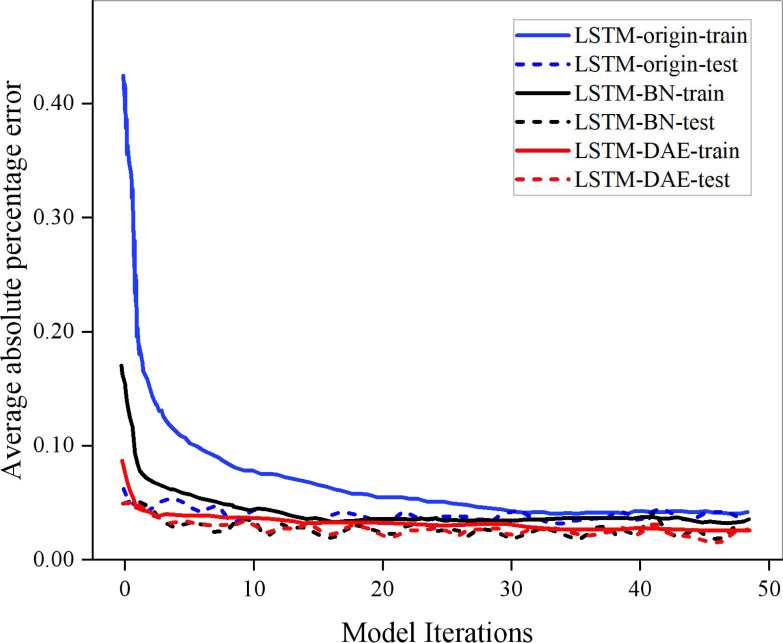
Comparison of the number of iterations and the error reduction effect of each model in the training set and test set.

The three models—the original LSTM-origin model, the improved LSTM-BN model, and the LSTM-DAE model—can be trained and tested using the same data set, and by using the same initial and iteration round numbers, it is possible to compare the number of iterations and error reduction of each model in the training and test sets. In Fig. 6, this is displayed. It can be seen from Fig. 6 that under the same training set and test set, the loss functions of the three model algorithms designed in this study for the SOC of the energy storage battery tend to be stable after multiple rounds of iterations. Because there is no longer a need to rely on the network initialization settings and learning rate, the training set and test set errors based on the original long short-term memory network are at their highest positions among the three models when the error tends to be stable. The iteration speed of the LSTM-BN model is substantially faster than that of the LSTM-origin model because of the model’s increased iteration efficiency, which also results in a decrease in error. The LSTM-BN technique is then further optimized using the DAE algorithm. The error is decreased to the lowest level among the three models with additional feature extraction and denoising optimization, and the fluctuation is also reduced to increase the model’s stability. The model’s resilience and generalizability are also gradually increased due to its optimization.

**Figure 7:**
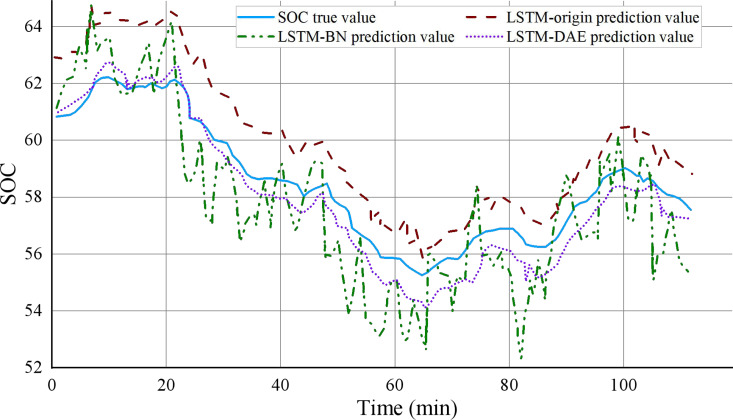
Comparison of predicted and true values of each model on the same data segment.

**Figure 8:**
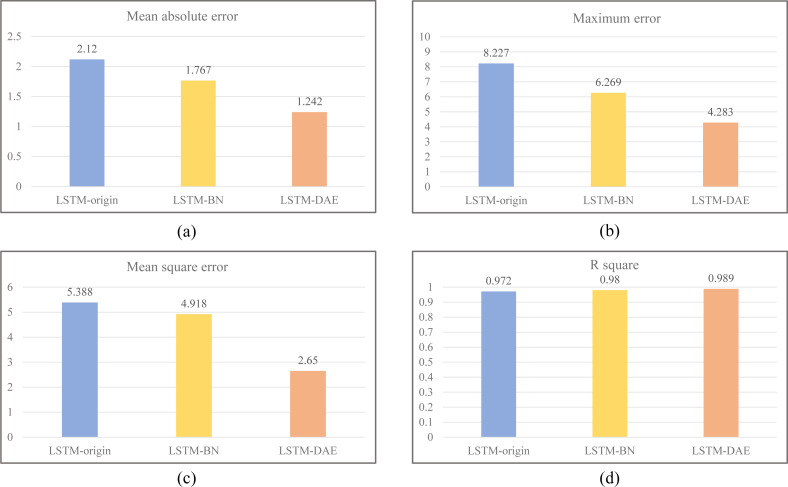
Comparison of the values of the four evaluation indicators of the three models designed in this study (including mean absolute error, maximum error, mean square error, and R square).

After the training set is finished and the test set is anticipated, Fig. 7 compares the accurate SOC with the SOC predicted by the model for the three models created in this study. Due to the significant number of samples in the test set, 115 sample points were chosen for this image to illustrate the true SOC value and the SOC value projected by each model to visually and unmistakably demonstrate the model’s predictive power. The expected value of the model’s LSTM origin may be seen in the Fig. 7 to have a similar trend to the actual value of SOC, but there is still a distinct inaccuracy. Between them, there is a substantial amount of variability. Last but not least, the DAE algorithm-optimized LSTM-DAE model can more closely track the real value with the least amount of error. Because of the sharply decreased fluctuation, the LSTM-DAE model provides the best resilience and predictive power. Figure 8 compares the values of the four evaluation indicators of the three models designed in this study (including mean absolute error, maximum error, mean square error, and R square). It can be seen that the performance indicators of the LSTM-DAE model are significantly better than other models, and the prediction accuracy and robustness have been substantially improved.

### Function test of wireless BMS monitoring alarm system

This research tests and verifies the functions of the wireless BMS monitoring and alarm system to ensure the BMS’s stable, reliable and accurate operation. The test plan mainly includes the safety performance test, system working condition test and routine performance test of the battery management system.

#### Safety performance test

**Figure 9:**
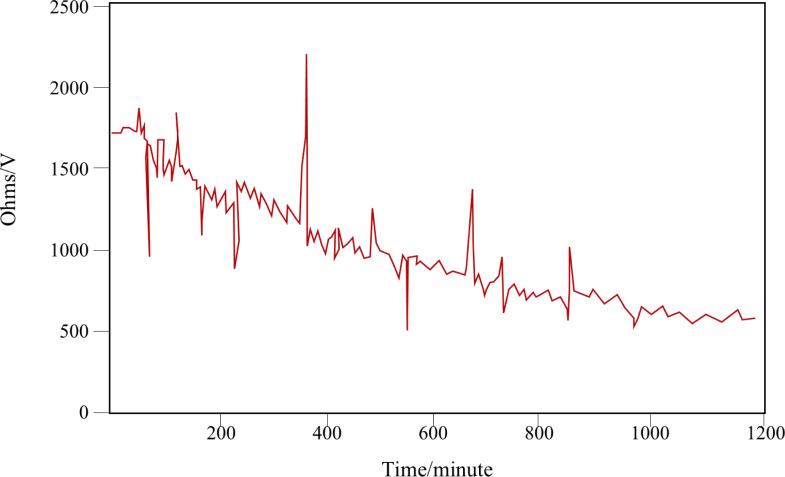
Diagram of insulation monitoring resistance.

The safety performance test of the battery management system is mainly aimed at the simulation test of insulation resistance. According to GB/T 18384-2015 Safety Requirements for Electric Vehicles, the insulation resistance of the BMS system shall not be less than 500 Ω/V (General Administration of Quality Supervision, 2015). This research monitors the change of insulation resistance by testing the insulation performance of the positive and negative poles of the high-voltage bus of the battery system. The results in Fig. 9 show that the system has high safety performance.

#### System working condition test

**Figure 10:**
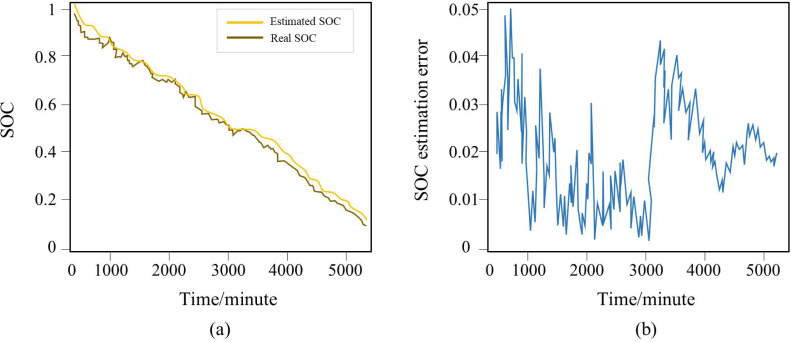
The (A) SOC estimation test and (B) SOC error chart.

Through the SOC estimation test, state quantity acquisition accuracy test, and fault diagnostic test, this research evaluates the system’s operating condition performance in an effort to decrease failure rates, enhance product quality and performance, and accelerate the development cycle of BMS. Figure 10 shows the SOC estimation test and SOC error chart. The test results show that when the SOC is>80%, the SOC estimation error can be maintained within 4%. When the SOC is<80%, the estimation error can be maintained within 5%, meeting the relevant requirements of the BMS design specification.

#### Routine performance test

**Figure 11:**
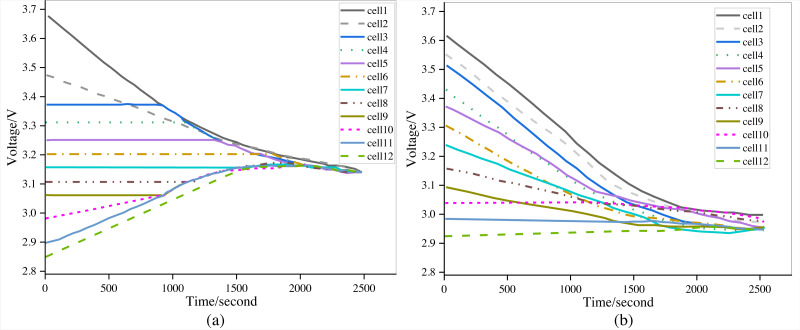
The (A) static equalization test and (B) discharge equalization test.

Static and discharge equalization tests are the critical components of the BMS hardware’s standard performance test. After the BMS is turned on, without starting the car model, static equalization sets various initial battery voltages and waits for automatic equalization. The BMS is configured to perform discharge testing under constant working conditions thanks to discharge equalization. Due to the equalization approach, the battery management system will carry out discharge equalization. This investigation chooses twelve batteries in the same BMS port for testing. In Fig. 11, the equalization image is displayed. Figure 11 shows the static equalization test and discharge equalization test. According to the test results, the voltage difference between each cell decreases with time, totally realizing the equalization function test and satisfying the equalization requirements.

## Conclusions

In light of the continuous growth of electric vehicles, power batteries have gained increasing prominence. In this regard, the present research delves into the theory and methodology of battery remote monitoring and alarm. It proposes a wireless BMS monitoring and alarm system based on Socket communication. The system enables researchers to remotely view the electric vehicle battery pack’s operation parameters and fault information and subsequently analyze the parameter data. The practical application significance of the system is paramount in advancing the research of battery management systems.

This study first applies the data-driven approach to enhance the estimation methodology for the battery’s state of charge in the energy storage system. The research suggests a short- and long-term memory network technique suitable for the time series data of battery characteristics to address the accuracy mistake in battery state of charge prediction. The model’s error stabilizes under 5% thanks to data annotation, data standardization, and processing of the LSTM input, forgetting, and output layers. The model is optimized in this work using the batch normalization procedure, considering the model’s strong reliance on initial set parameters, including learning rate, parameter initialization, weight attenuation coefficient, and Dropout ratio. The approach increases the training speed and improves the stability of the model.

Additionally, based on the estimation method of the energy storage battery’s state of charge, the study puts a wireless BMS monitoring and alarm system into practice. The system can be divided into four layers: business, algorithm, communication, and data. The business layer is primarily in charge of implementing interaction and request distribution between different system modules, storing business logic processing, distributing algorithm services to the algorithm layer, and achieving the corresponding functions *via* Socket communication and data persistence module interaction of the data layer. The study performs safety performance testing, system working condition tests, and routine performance tests for the system functions to guarantee BMS’s stable, dependable, and accurate functioning.

However, this study is based on a data-driven method to accurately estimate the state of battery charge accurately, mainly using voltage, current, temperature and other characteristics. Suppose other sensors can collect some electrochemical change characteristics inside the battery in the future. In that case, the change of battery SOC can be better characterized, which will improve the accurate prediction of battery SOC.

##  Supplemental Information

10.7717/peerj-cs.1345/supp-1 Supplemental Information 1 Code

10.7717/peerj-cs.1345/supp-2 Supplemental Information 2 Dataset for BMS monitoring

## Additional Information and Declarations

### Data Availability

The following information was supplied regarding data availability: The raw data and code are available in the Supplementary Files.

## References

[ref-1] Ali MU, Zafar A, Nengroo SH, Hussain S, Alvi MJ, Kim H-J (2019). Towards a smarter battery management system for electric vehicle applications: a critical review of lithium-ion battery state of charge estimation. Energies.

[ref-2] Baumann M, Rohr S, Lienkamp M (2018). Cloud-connected battery management for decision making on second-life of electric vehicle batteries.

[ref-3] Bizeray AM, Zhao S, Duncan SR, Howey DA (2015). Lithium-ion battery thermal-electrochemical model-based state estimation using orthogonal collocation and a modified extended Kalman filter. Journal of Power Sources.

[ref-4] Chen Z, Fu Y, Mi CC (2012). State of charge estimation of lithium-ion batteries in electric drive vehicles using extended Kalman filtering. IEEE Transactions on Vehicular Technology.

[ref-5] Chen Z, Xue Q, Xiao R, Liu Y, Shen J (2019). State of health estimation for lithium-ion batteries based on fusion of autoregressive moving average model and elman neural network. IEEE Access.

[ref-6] Ferguson JW, Baglino AD, Kalayjian NR (2014). Electromechanical pawl for controlling vehicle charge inlet access: US Patent 8.

[ref-7] Gabbar HA, Othman AM, Abdussami MR (2021). Review of battery management systems (BMS) development and industrial standards. Technologies.

[ref-8] Garcia P, Fernandez LM, Garcia CA, Jurado F (2009). Energy management system of fuel-cell-battery hybrid tramway. IEEE Transactions on Industrial Electronics.

[ref-9] General Administration of Quality Supervision (2015). Inspection and Quarantine of the Peoples Republic of China, Standardization Administration of the Peoples Republic of China. GB/T 18384.1-2015 Electrically propelled road vehicles-Safety specifications Part 1: On board rechargeable energy storage system (RESS) [S].

[ref-10] Hu C, Youn BD, Chung J (2012). A multiscale framework with extended Kalman filter for lithium-ion battery SOC and capacity estimation. Applied Energy.

[ref-11] Hua Y, Dong F (2022). How can new energy vehicles become qualified relays from the perspective of carbon neutralization? Literature review and research prospect based on the CiteSpace knowledge map. Environmental Science and Pollution Research.

[ref-12] Li X, Yuan C, Wang Z (2020). Multi-time-scale framework for prognostic health condition of lithium battery using modified Gaussian process regression and nonlinear regression. Journal of Power Sources.

[ref-13] Li Y, Wang C, Gong J (2016). A combination Kalman filter approach for State of Charge estimation of lithium-ion battery considering model uncertainty. Energy.

[ref-14] Li Z, Ma C, Deng B, Ou Y, Wang Y, Meng Z, Wang P, Fan J (2020). Dual time-scale co-estimation of state-of-charge and state-of-health for lithium-ion battery pack with passive balance control over whole lifespan based on particle filter.

[ref-15] Lin HT, Liang TJ, Chen SM (2012). Estimation of battery state of health using probabilistic neural network. IEEE Transactions on Industrial Informatics.

[ref-16] Liu Z, Li Z, Zhang J, Su L, Ge H (2019). Accurate and efficient estimation of lithium-ion battery state of charge with alternate adaptive extended Kalman filter and ampere-hour counting methods. Energies.

[ref-17] MacDougall RE, Bertolino JD, Rodden KL, Alger ET (2000). Lab testing of battery charge management systems for electric and hybrid electric vehicle battery packs to evaluate cycle life improvement.

[ref-18] Meng J, Ricco M, Luo G, Swierczynski M, Stroe D-I, Stroe A-I, Teodorescu R (2017). An overview and comparison of online implementable SOC estimation methods for lithium-ion battery. IEEE Transactions on Industry Applications.

[ref-19] Nandakumar R, Ponnusamy V, Mishra AK (2023). LSTM based spectrum prediction for real-time spectrum access for IoT applications. Intelligent Automation & Soft Computing.

[ref-20] Prabhu DK, Maheswari R, Vigneshwaran B (2022). Deep learning based power transformer monitoring using partial discharge patterns. Intelligent Automation & Soft Computing.

[ref-21] Shrivastava P, Soon TK, Idris MYIB, Mekhilef S (2019). Overview of model-based online state-of-charge estimation using Kalman filter family for lithium-ion batteries. Renewable and Sustainable Energy Reviews.

[ref-22] Strominger A (2014). On BMS invariance of gravitational scattering. Journal of High Energy Physics.

[ref-23] Wey CL, Jui PC (2013). A unitized charging and discharging smart battery management system.

[ref-24] Xing Y, He W, Pecht M, Tsui KL (2014). State of charge estimation of lithium-ion batteries using the open-circuit voltage at various ambient temperatures. Applied Energy.

[ref-25] Zhang H, Huang J, Hu R, Zhou D, Khan HURashid, Ma C (2020). Echelon utilization of waste power batteries in new energy vehicles: review of Chinese policies. Energy.

[ref-26] Zhang R, Xia B, Li B, Cao L, Lai Y, Zheng W, Wang H, Wang W (2018). State of the art of lithium-ion battery SOC estimation for electrical vehicles. Energies.

[ref-27] Zheng Y, Ouyang M, Han X, Lu L, Li J (2018). Investigating the error sources of the online state of charge estimation methods for lithium-ion batteries in electric vehicles. Journal of Power Sources.

[ref-28] Zhou W, Zheng Y, Pan Z, Lu Q (2021). Review on the battery model and SOC estimation method. Processes.

